# Shuangyu Tiaozhi Granule Attenuates Hypercholesterolemia through the Reduction of Cholesterol Synthesis in Rat Fed a High Cholesterol Diet

**DOI:** 10.1155/2019/4805926

**Published:** 2019-02-27

**Authors:** Jingjing Shi, Ruoqi Li, Yi Liu, Haifei Lu, Lu Yu, Fengxia Zhang

**Affiliations:** ^1^Shandong University of Traditional Chinese Medicine, Jinan 250000, China; ^2^Department of Neurology, Affiliated Hospital of Shandong University of Traditional Chinese Medicine, Jinan 250011, China

## Abstract

Shuangyu Tiaozhi Granule (STG) is composed of two kinds of Chinese medicinal herbs in* dioscorea*, which are used for managing cholesterol levels in patients with hypercholesterolemia in traditional Chinese medicine (TCM). However, the potential molecular mechanisms of administration of STG in hypercholesterolemia remain unknown. In this study, we investigated the effects of STG on hepatic cholesterol metabolism in high cholesterol (HC) diet-induced hypercholesterolemic rat models and simvastatin was used as a positive control. Male Sprague Dawley (SD) rats were fed general or HC diet, respectively. After 4 weeks of feeding, HC diet-induced hypercholesterolemic rats were fed HC diet, STG at 5% (w/w) or 10% (w/w) mixed in the HC diet, or HC diet combined with simvastatin gavages (4 mg·kg^−1^·d^−1^) for 4 or 8 weeks. STG treatment decreased body weight gain, liver weight ratio, serum lipids levels and hepatic lipids accumulation in rats fed a HC diet. Moreover, the effects of STG on decreasing body weight and lowering liver cholesterol levels were in dose- and time-dependent. Furthermore, STG or simvastatin treatment decreased the mRNA and protein levels of HMGCR and SREBP-2 in liver. The ACAT-2 and CYP7A1 mRNA expression were significantly decreased in HC diet supplemented with STG, while the mRNA levels of LDLR were markedly increased. STG attenuates hypercholesterolemia via inhibiting SREBP-2 signaling pathway activation and increasing hepatic uptake genes expression, providing a novel idea of TCM keeping cholesterol levels down for the clinical application.

## 1. Introduction

Hypercholesterolemia, an abnormal lipid metabolism disease, is the primary risk factor in the generation and development of coronary heart disease (CHD) [1, 2]. In particular, high concentration of circulating low density lipoprotein cholesterol (LDL-C) is often associated with the occurrence of myocardial infarction [3]. According to recent studies, CHD as a global epidemic of noncommunicable diseases is the leading cause of death [4], and the mortality rate of CHD in china increased from 16% to 24% in ten years [5, 6]. In addition, hypercholesterolemia is also linked to storks [7], nonalcoholic fatty liver disease (NAFLD) [8], and being overweight [9].

The sources of cholesterol in mammals are de novo biosynthesis from acyetyl-CoA, LDL receptor (LDLR)-mediated endocytosis from plasma and absorption from diet, in which 70% to 80% of cholesterol is synthesized by liver* in vivo* [10]. 3-hydroxy-3-methylglutaryl-CoA reductase (HMGCR) as the rate-limiting enzyme as well as the target of feedback regulation catalyzes HMG-CoA into mevalonate which is the key step of cholesterol synthesis. Moreover, the transcription of HMGCR gene is regulated by sterol regulatory element-binding protein-2 (SREBP-2) which is a master nuclear transcription factor [11]. According to clinical guidelines, statins targeted at reducing cholesterol synthesis are recommended as first-line treatment of patients with CHD caused by elevated cholesterol, especially LDL-C levels [12, 13]. However, even moderate- or high-intensity statins therapy also makes 20% of patients the LDL-C treatment goal of < 70 mg·dL^−1^ attainment [14]. And the therapy costs and side effects cannot be ignored [15, 16]. Therefore, there is urgently needed for focusing research hotspot on seeking new, safe, and effective cholesterol-lowering drugs.

Traditional Chinese medicine (TCM) with the multitarget and multipathway has advantage for therapying complex disease courses [17, 18]. Shuangyu Tiaozhi Granule (STG) composed of Shuyu and Bixie derives from Huazhuo Xingxue decoction which has been used to lower lipids level in clinical. Our previous studies have shown that STG can lower serum lipids levels in patients with hypercholesterolemia [19]. In the present study, we aim to investigate whether the effect of different doses and treatment times of STG, a traditional Chinese prescription, on body weight, serum lipids, and hepatic cholesterol levels is through regulating cholesterol synthesis pathway and LDLR-mediated cholesterol uptake in rat fed high cholesterol (HC) diet.

## 2. Materials and Methods

### 2.1. Animals

Fifty 6-week-old male Sprague Dawley (SD) rats weighting between 160 g and 180 g were purchased from Vital River Laboratory Animal Technology Co., Ltd (Beijing, China). The rats were housed under a 12 h light/dark cycle at a controlled temperature (23±2°C) in 50-60% humidity environment. And all rats were allowed free access to acquire energy and drinking water. All experiments were conducted in accordance with the Guide for the Care and Use of Laboratory Animals and approved by the Ethics Committee of Shandong University of Traditional Chinese Medicine.

### 2.2. The Preparation of STG

The components of STG include 60 g of Shuyu (Rhizoma Dioscoreae) and 18 g of Bixie (Dioscoreae Spongiosae Rhizoma). And the herbal concentrate-granules (1 g concentrate-granules = 20 g Chinese herbal pieces) were provided by Jiangyin Tian Jiang Pharmaceutical Co., Ltd. (Jiangsu, China), and were identified by Prof. Feng Li of Pharmacy College, Shandong University of Traditional Chinese Medicine. For* in vivo* studies, STG was delivered to Beijing Keaoxieli Feed Co., Ltd. (Beijing, China) in order to add 5% (w/w) or 10% (w/w) STG, respectively, to the HC diet (Table 1).

### 2.3. Induction of Hypercholesterolemia and Drug Treatment

After adapting to the environment for 1 week, all rats were fed with general diet or HC diet (Beijing Keaoxieli Feed Co., Ltd., Beijing, China) for 4 weeks. In many previous studies, the level of serum lipids of all rats elevated after being fed HC diet for 4 weeks [20]. All experimental rats were randomly divided into 5 groups (n = 10 per group): general diet group (control), HC diet group (HC), HC diet supplemented with high-dose STG group (10% (w/w) STG, HSTG), HC diet supplemented with low-dose STG group (5% (w/w) STG, LSTG), and HC diet supplemented with simvastatin group (simvastatin). Especially, simvastatin group was administered by intragastric of simvastatin (dissolved in saline to a dosage of 4 mg·kg^−1^·d^−1^, Hangzhou Moshadong Pharmaceutical Co., Ltd., Hangzhou, China). The body weights of rats were measured regularly every two weeks and the dosage of simvastatin was adjusted according to the weight of the rats. After being treated by STG or simvastatin for 4 or 8 weeks, rats were sacrificed to collect blood and liver samples. Part of liver tissue was fixed in 4% Paraformaldehyde for Hematoxylin and Eosin (H&E) staining and immunohistochemistry and the remaining part was frozen in liquid nitrogen and then stored at -80°C until further analysis.

### 2.4. Serum Analysis

Blood samples were collected from abdominal aorta. After placing for 1 h at room temperature, the blood samples were centrifuged at 1500 ×g for 10 min to obtain the supernatant. Serum samples were stored at -20°C and total cholesterol (TC), triglyceride (TG), low density lipoprotein-cholesterol (LDL-C), and high density lipoprotein-cholesterol (HDL-C) levels were measured by an automatic biochemical analyzer in clinical laboratory of the Affiliated Hospital of Shandong University of Traditional Chinese Medicine (Jinan, China).

### 2.5. Liver Lipids Assay

The liver lipids were measured using a tissue total cholesterol assay kit or tissue free cholesterol assay kit (E1015, E1016, Applygen Technologies Co., Ltd, Beijing, China) in accordance with the manufacturer's instructions. The cholesterol levels in liver were corrected by the protein concentrations.

### 2.6. H&E Staining

The liver tissues were embedded in paraffin and dissected into 5 *μ*m thick sections. Then the H&E stained sections were observed under a light microscopy (Olympus BX51, Japan).

### 2.7. Real-Time PCR

Total RNA was extracted from rat liver tissues with Trizol reagent (Invitrogen, Thermo Fisher Scientific, USA) following the manufacturer's instructions. RNA concentrations were analyzed spectrophotometrically (Nanodrop 2000c, Thermo Fisher Scientific, USA). According to the manufacturer's instructions, reverse transcription was performed with 5x All-In-One RT MasterMix kit (Abm, Canada), and real-time PCR was carried out using a SYBR Green PCR Master Mix kit (DBI Bioscience, Germany) in LC480 (Roche, Mannheim, Germany). All quantifications were performed using GAPDH as an endogenous control. The data was analyzed using 2^−△△CT^ method. Each sample was repeatedly tested three times. The primer sequences were listed in Table 2.

### 2.8. Protein Preparation and Western Blot

Samples of liver tissue were washed three times with cold phosphate-buffered saline (PBS) before being lysed in RIPA lysis buffer (Beyotime Biotechnology Company, Jiangsu, China) with phenylmethanesulfonyl fluoride (Beyotime Biotechnology Company, Jiangsu, China) on ice and centrifuged at 4°C, 6000 ×g for 20 min. BCA protein assay kit (Beyotime Biotechnology Company, Jiangsu, China) was used to measure protein concentrations. Briefly, total proteins (110 *μ*g) were separated on 10% SDS-polyacrylamide gel for electrophoresis and then transferred onto polyvinylidene fluoride (PVDF) membrane (0.45 *μ*m, Millipore, Billerica, MA, USA). After blocking with 5% non-fat milk for 1h at room temperature, the membrane was respectively incubated with the following primary antibodies: SREBP-2 (1:500, abcam, Burlingame, CA, USA, ab30682), HMGCR (1:3000, abcam, Burlingame, CA, USA, ab174830), or beta-actin (1:5000, Proteintech Group, Inc., wuhan, China) overnight at 4°C. The secondary antibodies conjugated to horseradish peroxidase (1:10000, Zhongshan Golden Bridge Biotechnology Co., Beijing, China) were used for incubation for one hour at room temperature. After washing three times in Tris-buffer saline containing 0.1% Tween 20 (TBST), the immunoreactive bands were detected with Immobilon Western Chemiluminescent HRP Substrate (Millipore Corporation, Billerica, USA) and exposed using Fluor Chem Q system.

### 2.9. Immunohistochemistry

Immunohistochemistry was performed using a universal two-step detection kit (PV9000, Zhongshan Golden Bridge Biotechnology Co, Beijing, China) to detect SREBP-2 protein expression in accordance with the manufacturer's instructions. Briefly, the liver sections were dewaxed, repaired antigen retrieval, and incubated with endogenous peroxide blocker for 10 minutes at room temperature and then incubated with the primary antibody (1:100, abcam, Burlingame, CA, USA, ab28482) overnight at 4°C. The protein expression was observed by laser-scanning microscopy (Zeiss Vert.A1, Carl Zeiss Canada).

### 2.10. Statistical Analysis

Data was represented as the mean ± standard deviation (SD). To compare multiple-group statistical differences, one-way ANOVA analysis followed by Fisher's least-significant difference (LSD) or Dunnett's test was used. The statistical analysis was performed using SPSS 23.0 software (SPSS, Inc., Chicago, IL, USA).* P*<0.05 was considered statistically significant.

## 3. Results

### 3.1. Body Weight and Liver Weight Ratio

**Figure 1:**
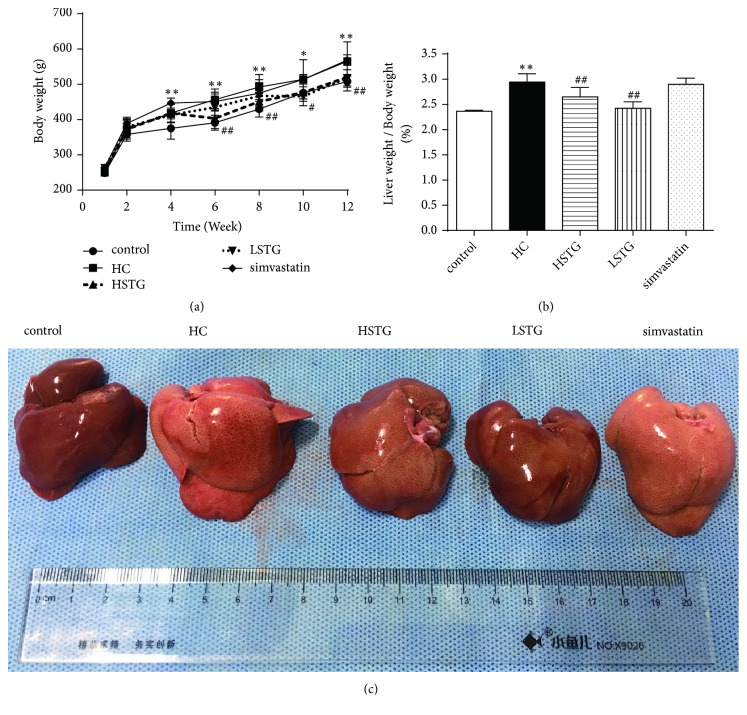
Effect of STG on body weight and liver weight ratio in rat fed a HC diet. (a) Body weight was measured every two weeks during 12 weeks of general or HC diet supplemented with or without STG or simvastatin. (b) Comparison of liver weight ratio after STG or simvastatin treatment for 8 weeks. Liver weight ratio is equal to liver wet weight/body weight × 100%. ^*∗*^*P *< 0.05, ^*∗∗*^*P *< 0.01 versus control group; ^#^*P *< 0.05, ^##^*P *< 0.01 versus HC group. (c) Photograph of liver tissues from rats treated by STG or simvastatin for 8 weeks. All data was shown as mean ± SD (n = 6 to 10).

Excessive accumulation of fat in the body is associated with overweight, hyperlipidemia and cardio cerebral vascular disease. Rapid body weight gains in rats fed HC diet without STG supplementation during the study process compared to control group (*P* < 0.01). Treatment with high dose of STG for 2 weeks, body weight was associated with a reduction compared to those in HC group (*P* < 0.01). While in LSTG group, the effect of body weight loss began to make sense at the end of 4 weeks of low dose of STG treatment, compared to HC group (*P* < 0.05) (Figure 1(a)). Compared with control group, liver weight ratios in HC group increased by 24.5% (*P *< 0.01), whereas liver weight ratio was reduced in both high and low dose of STG treatment for 8 weeks (Figure 1(b)). The liver appearance from HC group was bigger size than control group, and color was yellow and greasy by naked eye, while being treated with STG for 8 weeks alleviate these changes (Figure 1(c)). Conversely, there was no significant difference in body weight and liver weight ratios of rats between simvastatin group and HC group.

### 3.2. STG Ameliorates Hepatic Steatosis in HC Diet-Induced Hypercholesterolemic Rats

**Figure 2:**
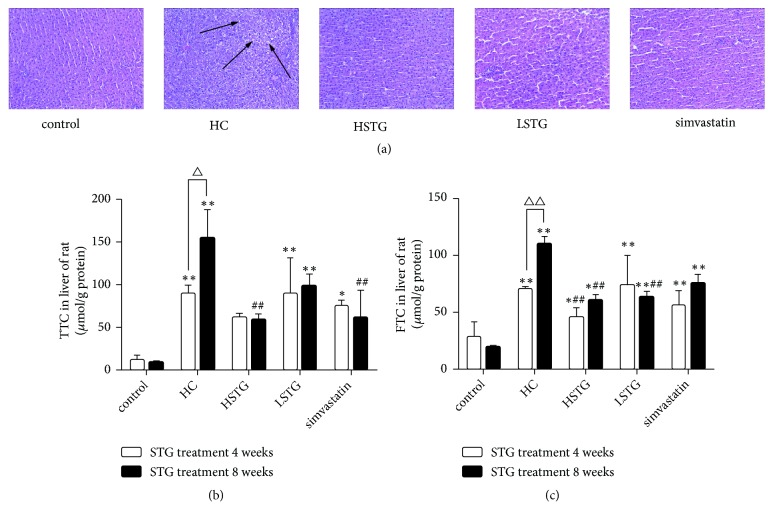
STG supplementation improved liver steatosis in rat fed a HC diet. (a) Liver histological changes were observed after 8 weeks of STG or simvastatin treatment. Liver sections stained with H&E (magnification, ×200). (b-c) Hepatic total cholesterol (TTC) and free cholesterol (FTC) were detected after STG or simvastatin treatment for 4 or 8 weeks. ^*∗*^*P *< 0.05, ^*∗∗*^*P *< 0.01 versus control group; ^##^*P *< 0.01 versus HC group; ^△^*P *< 0.05, ^△△^*P* < 0.01 versus STG treatment for 4 weeks. The arrows point to fat droplet. All data were shown as mean ± SD.

To observe histopathology of liver, liver samples were obtained after HC diet mixed 5%, 10% STG or simvastatin treatment for 8 weeks. H-E staining showed obvious lipid accumulation in the hepatocytes filled with small vacuoles in HC group. Among HSTG, LSTG, and simvastatin groups, hepatocytes were arranged regularly and few fat vacuoles can be seen (Figure 2(a)). Consistently, TTC and FTC levels were significantly increased after HC diet feeding for 4 or 8 weeks compared to control group, and 8 weeks of HC diet feeding increased more significantly (*P* < 0.01). Compared to HC group, TTC levels were decreased by the treatment of high dose of STG for 8 weeks (*P* < 0.01). However, high dose of STG treatment for 4 or 8 weeks and low dose of STG treatment for 8 weeks were associated with a significant decrease in FTC levels (Figures 2(b) and 2(c)).

### 3.3. STG Regulates Serum Lipids in HC Diet-Induced Hypercholesterolemic Rats

**Figure 3:**
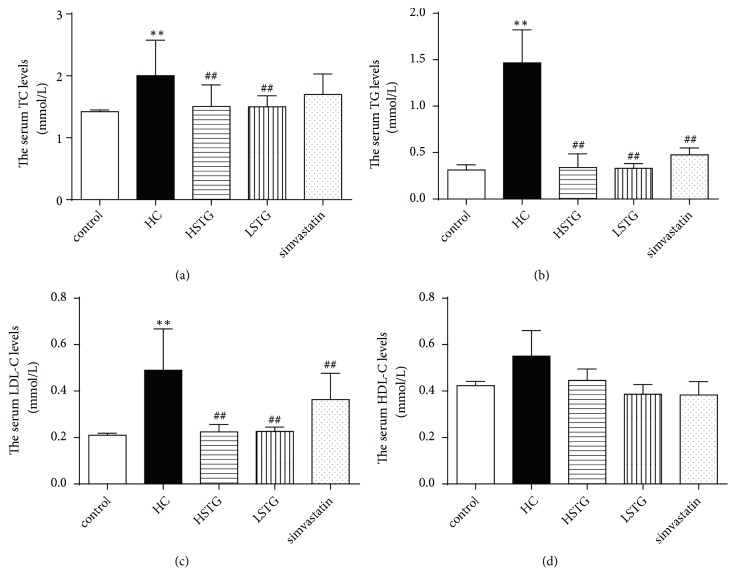
STG supplementation decreased serum lipid levels in rat fed a HC diet. The serum TC, TG, LDL-C, and HDL-C levels were detected after STG or simvastatin treatment for 4 or 8 weeks. TC, total cholesterol; TG, triglyceride; LDL-C, low density lipoprotein-cholesterol, HDL-C, high density lipoprotein-cholesterol. ^*∗∗*^*P *< 0.01 versus control group; ^##^*P *< 0.01 versus HC group. All data were shown as mean ± SD (n = 9 to 10).

Compared with control group, HC diet markedly increased serum TC, TG, and LDL-C levels (*P* < 0.01). As expected, HSTG group and LSTG group showed a reduction in TC, TG, and LDL-C levels compared with HC group. Rats fed a HC diet supplemented with simvastatin had lower TC, TG, and LDL-C levels, while only TG and LDL-C levels were significantly decreased compared to those in HC group (Figures 3(a)–3(c)). The HDL-C levels were not significantly changed among HC, HSTG, and LSTG as well as simvastatin group (Figure 3(d)).

### 3.4. STG Decreases Cholesterol Synthesis through Suppressing Expression of SREBP-2 and HMGCR in Liver

**Figure 4:**
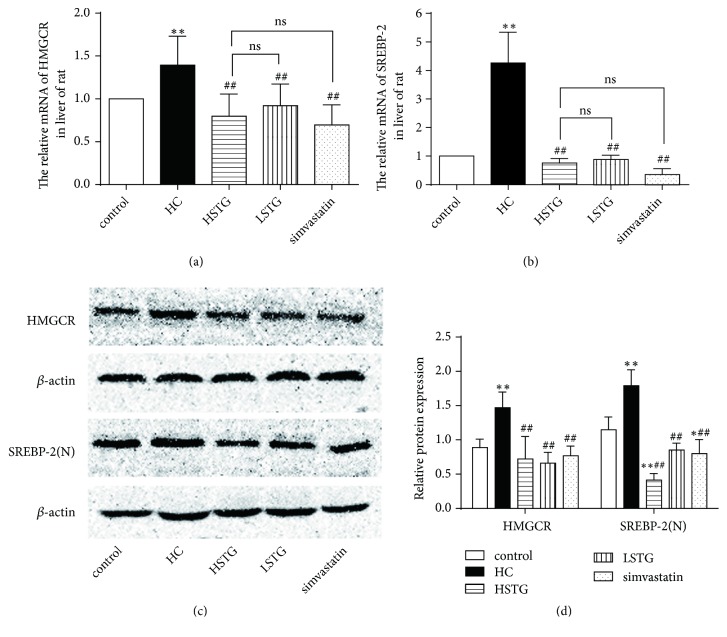
Effect of STG on expression of HMGCR and SREBP-2 in rat fed a HC diet. Relative mRNA expression (a-b) and protein expression (c-d) of HMGCR and SREBP-2 were analyzed after STG or simvastatin treatment for 8 weeks. The independent experiment was repeated three times. SREBP-2 (N), nuclear SREBP-2. ^*∗*^*P *< 0.05, ^*∗∗*^*P *< 0.01 versus control group; ^##^*P *< 0.01 versus HC group; ^ns^*P *> 0.05 versus HSTG group. All data were shown as mean ± SD.

**Figure 5:**
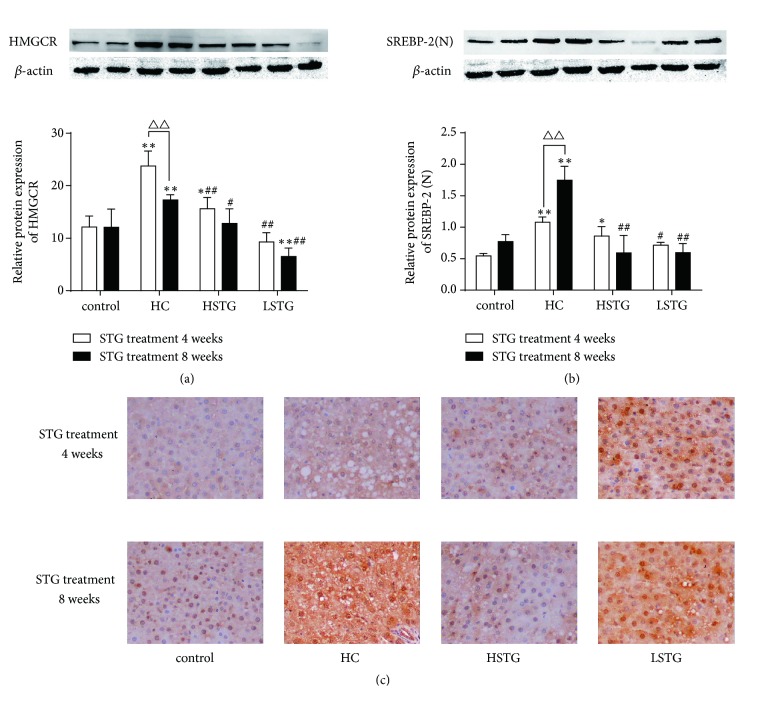
Different treatment times of STG on protein expression of HMGCR and SREBP-2 in rat fed a HC diet. (a-b) Western blotting was performed to detect HMGCR and SREBP-2 protein levels in rat treated by high or low dose of STG for 4 or 8 weeks. ^*∗*^*P *< 0.05, ^*∗∗*^*P *< 0.01 versus control group; ^#^*P *< 0.05, ^##^*P *< 0.01 versus HC group; ^△△^*P *< 0.01 versus STG treatment for 4 weeks. SREBP-2 (N), nuclear SREBP-2. (c) The expression of SREBP-2 in rat liver tissue was detected by immunohistochemistry after STG treatment for 4 or 8 weeks. All data were shown as mean ± SD.

The mRNA levels of HMGCR and SREBP-2 were increased in HC group compared to control group (*P* < 0.01). When treated with STG or simvastatin for 8 weeks, HMGCR and SREBP-2 mRNA expression were reduced in HSTG group (43%, 82%, respectively), in LSTG group (34%, 79%, respectively) and in simvastatin group (50%, 92%, respectively). However, the effects among HSTG, LSTG, and simvastatin group were no significant difference (*P* > 0.05) (Figures 4(a) and 4(b)). Consistently, compared with control group, the expressions of HMGCR and SREBP-2 protein were increased in HC group (*P* < 0.01), and this increase was decreased after STG or simvastatin treatment for 8 weeks (Figures 4(c) and 4(d)). In either HC diet fed for 4 or 8 weeks, the HMGCR expression was increased (*P* < 0.01). When being treated with high or low dose of STG, HMGCR expression was decreased by 34% or 61% at the end of the 8 weeks and 26% or 62% at the end of the 12 weeks (Figure 5(a)). Similarly, STG reduced SREBP-2 protein expression in HSTG and LSTG group after STG treatment for 4 or 8 weeks (Figure 5(b)). Furthermore, immunohistochemical staining showed that lots of stained buffy bundles were observed in cell nucleus and cell cytoplasm in HC group. SREBP-2 protein was feeble stained in high and low dose of STG treatment group compared to HC group (Figure 5(c)).

### 3.5. Expression of Cholesterol Metabolism Related Genes

**Figure 6:**
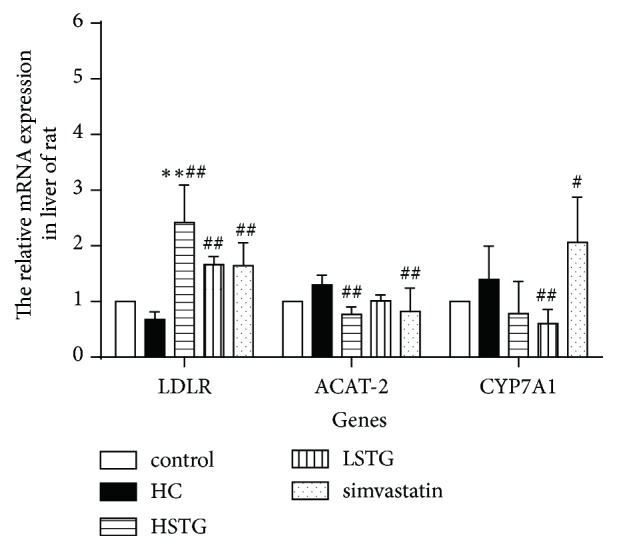
Effect of STG on mRNA expression of cholesterol metabolism related genes in rat fed a HC diet. Liver samples were obtained after STG or simvastatin treatment for 8 weeks. LDLR, ACAT-2, and CYP7A1 expression were detected by real-time PCR. ^*∗∗*^*P *< 0.01 versus control group; ^#^*P *< 0.05, ^##^*P *< 0.01 versus HC group. All data were shown as mean ± SD.

To investigate the effect of STG on cholesterol metabolism, we examined the mRNA expression of LDLR, ACAT-2, and CYP7A1 in liver using real-time PCR after STG treatment for 8 weeks (Figure 6). Compared with control group, there was a slight decrease in LDLR mRNA levels in HC group. Unlike the decrease in HMGCR and SREBP-2 mRNA levels, the mRNA levels of LDLR were significantly increased in HSTG, LSTG, and simvastatin group (257%, 145%, and 142%, respectively) compared to HC group. However, high dose of STG and simvastatin treatment were associated with a decrease (41%, 37%, respectively) in the mRNA expression of ACAT-2 compared to HC group. And CYP7A1 mRNA expression was decreased by 57% in LSTG group.

## 4. Discussion

**Figure 7:**
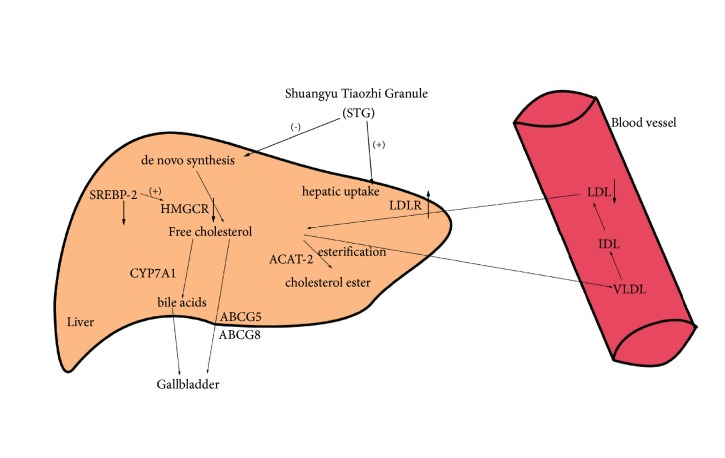
Schematic diagram of molecular mechanisms of Shuangyu Tiaozhi Granule (STG) attenuates hypercholesterolemia in rat fed HC diet. STG reduces contents of plasma cholesterol through the inhibition of cholesterol synthesis pathway and promotion of hepatic cholesterol uptake. STG treatment downregulated HMGCR and SREBP-2 expression and increased LDLR levels, whereas circulating LDL-C was uptake from plasma. HMGCR, 3-hydrox-3-methylglutaryl-CoA reductase; LDLR, low-density lipoprotein receptor; SREBP-2, sterol regulatory element-binding protein-2; ACAT-2, acyl coenzyme A-cholesterol acyltransferase-2; CYP7A1, cholesterol 7-*α*-hydroxylase; ABCG5, ATP-binding cassette transporter G5; ABCG8, ATP-binding cassette transporter G8; (+) promotion, (-) inhibition, ↑ represents upregulation, ↓ represents downregulation.

In the present study, STG treatment significantly ameliorated lipid metabolism disorder through the inhibition of cholesterol synthesis pathway and upregulation of LDLR-mediated cholesterol uptake (Figure 7). This research indicated that STG may provide a novel supplementary therapy for hypercholesterolemia and represent a molecular mechanism of TCM for the treatment of hypercholesterolemia.

Animal fed a HC diet to establish model of hypercholesterolemia mimicking human pathophysiology is common [20–22]. In current study, rats in HC group were associated with a significant increase both in TC and in LDL-C levels. Type 2 diabetic rats induced by high fat diet combined with streptozotocin (HFD-STZ) have shown body weight decreased, after administration of Dio which is an extract from dioscorea [23]. Consistent with these reports, the results demonstrated that supplementation with STG at both 5% and 10% concentrations can lose body weight gain as well as decrease liver weight ratio compared with hypercholesterolemia model in present study. Hepatocyte lipids accumulation is associated with hepatic steatosis and NAFLD which can increase risk of cardiovascular disease [24, 25]. Previous studies have shown that Dio has the effect of lowering lipids in liver [26, 27]. Consistent with these studies, the current study showed that administration of STG reduced liver lipids accumulation in HC-induced hypercholesterolemic rats. With the above studies, it suggested that administration of STG reduces excessive accumulation of lipids not only in the body but also in liver and thus loses body weight gain and ameliorates hepatic steatosis. Furthermore, this is the first time demonstration that STG supplementation attenuated lipid accumulation in body and liver with a time-and dose-dependent manner.

Dyslipidaemia especially hypercholesterolemia is one of the most important and controllable risk factors for CHD [2, 28]. As we all know, the hypocholesterolemic effect of statins has been fully confirmed in clinical applications, and using statins to lower cholesterol levels is a routine treatment for CHD [13]. Furthermore, its cholesterol modulating function is due to the effects of inhibition of HMGCR activation to reduce cholesterol synthesis [29]. Here, the changes of serum lipid content in rat fed a HC diet supplemented with STG in the present study were similar to those treated by simvastatins, suggesting that STG may be a novel cholesterol-lowering medicine as a useful adjunct therapy for hypercholesterolemia. However, the potential molecular mechanism of STG lowering serum and liver lipids is unclear. There is a precise regulatory mechanism to maintain cholesterol homeostasis* in vivo*, in which increasing cholesterol synthesis and decreasing uptake LDL-C from blood are main reasons for hypercholesterolemia [30]. Hence, lowering circulating LDL-C levels are first and major steps for treatment of atherosclerosis. Prior research coincides with our results that Dio attenuates aortic atherosclerosis via increasing cholesterol efflux and inhibiting macrophage miR-19b expression, providing evidence for Dio reducing cholesterol levels from macrophage cholesterol metabolism pathway [31]. In terms of hypocholesterolemic effects, several researches have demonstrated that Dio ameliorated lipid profiles through the AMPK and LXR pathways [26], the AMPK–ACC pathway [32], and absorption of cholesterol from intestine [33]. Our data showed that STG decreased the mRNA and protein levels of HMGCR, SREBP-2, and the mRNA expression of ACAT-2 and CYP7A1 in the liver. While the mRNA levels of LDLR were increased by STG treatment for 8 weeks. The data indicated that the hypocholesterolemic effects of STG were associated with cholesterol synthesis genes reduction and hepatic uptake genes increment.

From acetyl coenzyme A to cholesterol, there are more than 30 steps enzymatic reactions in which HMGCR catalyzes the conversion of HMG-CoA into metholvalic as a rate-limiting step. Hae-Ki Min [25] and Yujie Li [34] suggested that HC diet stimulates HMGCR activation which is closely associated with both hepatic and serum cholesterol. On the contrary, other research provides evidence that HMGCR expression was reduced in rat fed a HC diet [35, 36]. However, in our study, after 4 weeks of HC diet, both mRNA and protein expression of HMGCR were significantly increased compared to control group. Consequently, HMGCR overexpression is the main cause of elevated serum LDL-C level and cholesterol accumulation in liver. In the current study, both supplementations of 5% or 10% STG were associated with a decrease in expression of HMGCR. SREBP-2, a nuclear transcription factor, plays an important role in regulation of cholesterol synthesis by controlling the expression of HMGCR in liver [37]. It has been confirmed that HC diet induces SREBP-2 precursors releasing transcriptional active N-terminu entered the nucleus to guide the synthesis of HMGCR mRNA [38]. Similarly, the mRNA and protein levels of SREBP-2 were decreased in both high and low doses of STG treatment for 4 or 8 weeks. The data suggesting that STG treatment improved serum lipids profile by inhibiting SREBP-2 and HMGCR levels and thus reduced cholesterol synthesis.

LDLR plays a crucial role in the maintenance of cholesterol homeostasis, and LDLR mediated LDL-C uptake is the most important way of removing serum cholesterol in the body [39]. LDLr^−/−^ mice fed a high fat diet display a high cholesterol levels in serum and liver and thus were used to develop atherosclerosis and fat liver model [40–42]. We examined the mRNA of LDLR in the liver, and results suggested that STG can significantly elevate LDLR mRNA levels. In addition to downregulation of cholesterol synthesis genes, this study also showed that STG decreased ACAT-2 mRNA expression and then reduced cholesterol esterification. Hepatic ACAT-2 is involved in apoB-containing lipoproteins (VLDL, LDL) cholesterol packaging and deletion of hepatic ACAT-2 resulted in reduction of VLDL cholesterol [43]. Most of VLDL turned into LDL after entering the blood. Here, we showed that STG decreased hepatic ACAT-2 mRNA expression, thus contributing to reduction of serum LDL-C levels.

In TCM, spleen deficiency and damp abundance are often responsible for dyslipidemia. Deferring from Dio, a main active component isolated from dioscorea herbs [44, 45], STG treatment reduces serum and liver cholesterol from multiple targets and multiple levels with combination of shuyu and bixie. In conclusion, the results of the present study demonstrated that STG attenuates hypercholesterolemia and reduces hepatic accumulation of cholesterol in rat fed a HC diet. The cholesterol-lowering actions of STG were downregulated cholesterol synthesis genes and increased the clearance of plasma LDL-C. These results indicated that STG is an effective treatment to lower cholesterol levels and thus slow down the occurrence and development of CHD. Our research has only revealed STG supplementation lower cholesterol levels being time- and dose-dependent in two concentrations. And further studies of the inclusion of setting concentration gradient of STG and adding the clinical parts may be helpful to explore dose-effect relationship of STG in attenuating hypercholesterolemia.

## Figures and Tables

**Table 1:** Composition and nutrition components of experimental diets. The nutrition components are expressed as g/100 g.

Ingredients (%)	general diet	high cholesterol diet		
	control	HC	HSTG	LSTG
general diet	100	97.7	87.7	92.7
(i) crude protein	20	19.54	17.54	18.54
(ii) crude lipid	4.5	4.4	3.95	4.17
(iii) crude fiber	3.7	3.61	3.24	3.43
(iv) crude asb	6.53	6.38	5.73	6.05
(v) moisture	11	10.75	9.65	10.2
(vi) premix	54.27	53.02	47.59	50.31
cholesterol	-	2	2	2
sodium cholate	-	0.3	0.3	0.3
STG	-	-	10	5

**Table 2:** The primer sequences for real-time PCR.

Gene	Sequence (5′ - 3′)	
HMGCR	Forward primer	CCTCCATTGAGATCCGGAGG
	Reverse primer	AAGTGTCACCGTTCCCACAA
SREBP-2	Forward primer	GGCTGTCGGGTGTCATGGG
	Reverse primer	CTGTTCTCATCCATCGCCCAG
LDLR	Forward primer	TCACTGAAGCGCAAGGAGGA
	Reverse primer	ATGTCACCTTGGACTTGGGA
ACAT-2	Forward primer	TATGCACGGCCCCCAATATG
	Reverse primer	CCACAAGACAACAAGCAAGCA
CYP7A1	Forward primer	CTCTAAATGCCCTGCAGATGA
	Reverse primer	GGCACGGCTAATGATTCTCT
GAPDH	Forward primer	TCTCTGCTCCTCCCTGTTCT
	Reverse primer	ATCCGTTCACACCGACCTTC

## Data Availability

All data used or analyzed of this study are available upon request by contact with the corresponding author.
